# A Case of Spontaneously Resolving Cushing Disease

**DOI:** 10.1210/jcemcr/luaf131

**Published:** 2025-07-10

**Authors:** Aongus O’Brolchain, Simon Ryder, Kathryn Berkman

**Affiliations:** Logan Endocrine and Diabetes Service, Meadowbrook Medical Centre, Meadowbrook, QLD 4131, Australia; Griffith University, 58 Parklands Drive, Southport, QLD 4215, Australia; Logan Endocrine and Diabetes Service, Meadowbrook Medical Centre, Meadowbrook, QLD 4131, Australia; Logan Endocrine and Diabetes Service, Meadowbrook Medical Centre, Meadowbrook, QLD 4131, Australia

**Keywords:** Cushing, hypercortisolism, pituitary

## Abstract

We report a case of spontaneous resolution of Cushing disease (CD). A 20-year-old female individual, previously healthy, was referred for investigation of galactorrhea and a prolactin level of 31 ng/mL (<20 ng/mL) (SI: 31 µg/L, <20 µg/L). A magnetic resonance imaging (MRI) scan revealed an 11 mm pituitary lesion. Cabergoline was commenced to address symptomatic hyperprolactinemia. Over the following months, signs of hypercortisolism evolved. Initial tests revealed a positive 1-mg dexamethasone suppression test (DST), prompting further evaluation. Midnight salivary and 24-hour urinary free cortisol tests confirmed hypercortisolism, consistent with a pituitary source which was confirmed with a peripheral corticotropin-releasing hormone (CRH) stimulation test. While cabergoline successfully resolved the galactorrhea, an MRI scan 6 months later showed an increase in the pituitary lesion’s size, leading to neurosurgical referral. However, a preoperative MRI surprisingly revealed complete resolution of the pituitary mass, which coincided with the normalization of hypercortisolism symptoms and laboratory results. This rare case of spontaneous remission, while possibly influenced by cabergoline or undetected pituitary apoplexy, contributes valuable insights into the unpredictable nature of CD and its treatment.

## Introduction

Cushing disease (CD) results from hypercortisolism secondary to the production of adrenocorticotropin (ACTH) from a pituitary adenoma or carcinoma. Definitive treatment of CD is transsphenoidal surgery at an experienced pituitary center with success rates approaching 80%, although up to 30% of patients will require additional treatments [1]. Where surgery is contraindicated or otherwise not feasible, medical treatment can be offered to control symptoms of hypercortisolism, but intent is not curative. We present a case of spontaneous remission of CD.

## Case Presentation

In February 2023, a twenty-year-old female individual was referred for evaluation of spontaneous galactorrhea, with onset 2 months earlier. There was no significant past medical history, and the only prescription or over-the-counter medication was a combined oral contraceptive pill (COCP) (levonorgestrel 150 mcg/ethinylestradiol 30 mcg). External investigations included a normal cortisol (20 µg/dL [SI: 552 nmol/L]) (reference range, 5-23 µg/dL [SI: 140-640 nmol/L]) mildly elevated prolactin at 31 ng/mL (SI: 31 μg/L), reference range (<20 ng/mL) [SI: <20 μg/L]) and a magnetic resonance imaging (MRI) brain scan demonstrating an 11-mm pituitary lesion with suprasellar extension, but with no encroachment on the optic chiasm. Computerized perimetry by an external optometrist was normal. The remainder of the pituitary profile was not yet assessed.

At her initial endocrinology consultation, there were no symptoms of pituitary excess or deficiency. Her menses had regularized following a single, irregular period at the onset of symptoms.

On examination, blood pressure was 112/80 mmHg with no orthostatic drop. Specifically, there were no cushingoid or acromegalic features, no evidence of virilization, and the visual fields were normal to confrontation.

A provisional diagnosis of nonfunctioning pituitary macroadenoma with hyperprolactinemia secondary to “stalk effect” was made and low-dose cabergoline (500 mcg weekly), was commenced in March 2023.

At follow-up 6 months later, galactorrhea had resolved. Cabergoline was well-tolerated. The COCP was ceased by the patient 2 weeks prior. Repeat pituitary profile, shown in Table 1, included a mildly raised insulin-like growth factor 1 (IGF-1) level. Another appointment was booked for repeat blood tests and review of the surveillance MRI in 6 weeks.

**Table 1.** Biochemical investigations

Test	Reference Range	August 2023	September 2023	February 2024	March 2024	December 2024
Midnight salivary cortisol	0-3.8 ng/mL SI: 0-5 nmol/L	2.1 ng/mL 2.8 nmol/L	5.2 ng/mL 6.8 nmol/L	12.2 ng/mL 16.0 nmol/L	0.3 ng/mL 0.4 nmol/L	<2.3 ng/mL <3 nmol/L
24-hour urinary free cortisol	<10 µg/dL SI: <280 mmol/L	—	13 µg/dL 360 nmol/24 h	18 µg/dL 494 nmol/L	2.8 µg/dL 76 nmol	—
Cortisol:creatinine ratio		—	36	38	<8	—
				Oct 2023	Feb 2024	Nov 2024
Basal cortisol	8-24 μg/dL SI: 220- 660 nmol/L	22 µg/dL 610 nmol/L	18.5 µg/dL 510 nmol/L	40 µg/dL 420 nmol/L	7 µg/dL 180 nmol/L	16 µg/dL 446 nmol/L
Basal ACTH	0.3-3.4 ng/dL SI: 1.1- 11.1 pmol/L	3.7 ng/dL 12.3 pmol/L	3 ng/dL 10.0 pmol/L	—	26 ng/dL 5.7 pmol/L	—
Post-DST cortisol	<1.8 μg/dL SI: <50 nmol/L	17 µg/dL 470 nmol/L	8.3 µg/dL 230 nmol/L	—	—	—

Pituitary profile	Reference range	March 2023	August 2023	August 2023	February 2024
Prolactin	<20 ng/mL SI: <20 μg/L	31 ng/mL 31 µg/L	<1.3 ng/mL <1.3 µg/L	<1.3 ng/mL <1.3 µg/L	3.2 ng/mL 3.2 µg/L
FSH		6.4 IU/L	—	8 IU/L	8 IU/L
LH		1.9 IU/L	—	8 IU/L	6 IU/L
Estradiol (E2)	(pg/dL) [SI: pmol/L]	—	—	270 pg/dL 100 pmol/L	6000 pg/dL 220 pmol/L
TSH	(mIU/L) [0.55-4.78]	—	—	1.5 mIU/L	69 mIU/L
IGF-1	130-320 µg/L SI: 17-42 nmol/L	375 µg/L 49 nmol/L	474 µg/L 62 nmol/L	413 µg/L 54 nmol/L	168 µg/L 22 nmol/L

Abbreviations: FSH, follicle-stimulating hormone; IGF-1, insulin-like growth factor 1; LH, luteinizing hormone; TSH, thyrotropin (thyroid-stimulating hormone).

At this point, IGF-1 was persistently raised (see Table 1) and an MRI demonstrated interval growth of the pituitary macroadenoma. In August 2023, the patient reported weight gain of almost 8 to 10 kg in addition to worsening acne over the previous 2 months, which she had attributed to cessation of the COCP. The patient reported long-standing headaches occurring once or twice per week, which did not require pharmacotherapy, and no visual disturbance was reported. On examination, there was facial plethora and moon facies, but no interscapular fat pads, abdominal striae, or proximal myopathy, and the visual fields were again assessed to be normal. There were no clinical signs of acromegaly, but in view of the persistently raised IGF-1 level, a growth hormone (GH) suppression test was performed in August 2023 (Table 2). Following 75 g of oral glucose, the nadir GH level was 0.5 ng/mL (SI: 0.5μg/L) (reference range 0.05-8 ng/mL [SI: 0.05-8 μg/L]), excluding GH excess.

**Table 2.** Dynamic tests

Dynamic testing*^a^*	Reference range					
GH suppression*^b^* test (August 1, 2023)		0 minutes	30 minutes	60 minutes	90 minutes	120 minutes
IGF-1	130-320 µg/L SI: 17-42 nmol/L	436 µg/L 57 nmol/L	—	—	—	—
GH	0.05-8 ng/mL SI: 0.05-8 µg/L	8.55 ng/mL 8.55 µg/L	2.89 ng/mL 2.89 µg/L	0.92 ng/mL 0.92 µg/L	0.50 ng/mL 0.50 µg/L (nadir)	1.42 ng/mL 1.42 µg/L

CRH stimulation test*^c^* (September 29, 2023)	Ref Range	−5 minutes	−1 minute	+15 minutes		+30 minutes	+45 minutes	+60 minutes
ACTH	10-50 pg/mL SI: 2-11 pmol/L	52 pg/mL 11.5 pmol/L	40 pg/mL 8.8 pmol/L	110 pg/mL 24.2 pmol/L		84 pg/mL 18.5 pmol/L	79 pg/mL 17.4 pmol/L	54 pg/mL (11.9 pmol/L)
Cortisol		130 µg/L 361 nmol/L	420 nmol/L 152 µg/L	544 nmol/L 197 µg/L		596 nmol/L 216 µg/L	610 nmol/L 221 µg/L	584 nmol/L (212 µg/L)
4 mg IV DST*^d^* (October 3, 2023)		−60 minutes	0 minutes	+3 hours	+4 hours	+5 hours	+23.5 hours	+24 hours
Cortisol		174 µg/L 479 nmol/L	125 µg/L 344 nmol/L	59 µg/L 163 nmol/L	36 µg/L 100 nmol/L	27 µg/L 75 nmol/L	132 µg/L 365 nmol/L	111 µg/L 307 nmol/L

Abbreviations: ACTH, adrenocorticotropic hormone; DST, dexamethasone suppression test; GH, growth hormone.

^*a*^Procedures, cutoffs, and interpretation are based on the Harmonisation of Endocrine Dynamic Testing for Adults (HEDTA), The Endocrine Society of Australia and The Australasian Association of Clinical Biochemists, Australia, 2021 [1].

^*b*^2014 Endocrine Society Clinical Practice Guideline recommended using a universal cutoff of failure to suppress below <1.0 ug/L for acromegaly diagnosis [1].

^*c*^There is no universal agreement on blood test interval and cutoff. Pituitary Cushing disease is more likely than ectopic ACTH production if: Peak ACTH increment of >50% from mean basal values (sensitivity 86%, specificity 90%). increase in peak cortisol concentration ≥30% above the mean basal values (sensitivity 61%, specificity 70%) [1].

^*d*^Diagnosis of Cushing disease based on Day 2 serum cortisol level (mean of +23.5 hours and +24 hours cortisol values) >130 nmol/L or >20% of baseline cortisol (Day 1 at −60 minutes) [1].

Screening for Cushing syndrome was undertaken with a 1-mg overnight dexamethasone suppression test (DST), which did not suppress morning cortisol (see Table 1). Next, serial, timed urinary free and midnight salivary cortisol measurements were collected and a 4-mg, 2-day DST was arranged (Table 1). Once hypercortisolism was demonstrated, a peripheral corticotropin-releasing hormone (CRH) stimulation test was performed to localize the source of ACTH excess. In addition, a 4-mg intravenous DST was performed. Protocols for dynamic testing procedures, cutoffs/reference ranges and interpretation were derived from the Harmonisation of Endocrine Dynamic Testing for Adults (HEDTA), The Endocrine Society of Australia and The Australasian Association of Clinical Biochemists, Australia, 2021 [2]. Results of these tests are shown in Table 2. Based on these results (>100% increase from baseline ACTH and >50% increase in baseline cortisol following peripheral CRH) in combination with an evolving clinical picture, a diagnosis of CD was made. The case was discussed at a neurosurgical multidisciplinary meeting in September 2023, and the patient was referred for transsphenoidal pituitary surgery.

**Figure 1.**
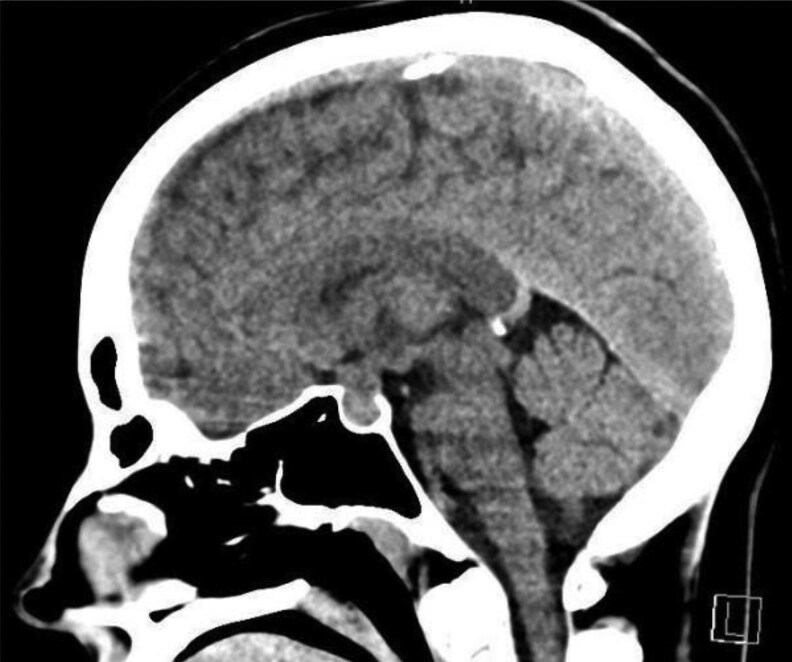
Figure shows a noncontrast computerized tomography (CT) in the sagittal plane of the brain at time of presentation with vomiting, photophobia, and headache in October 2023.

Subsequently in October 2023, the patient presented to the emergency department complaining of right-sided, retroorbital headache and photophobia, with associated vomiting. There was normal visual acuity and no evidence of ophthalmoplegia. Visual fields were not assessed, but a noncontrast computerized tomography (CT) scan of the brain was unremarkable (Fig. 1), and the pituitary lesion was stable in morphology and dimensions (within the limitations of intermodal assessment) and with no evidence of hemorrhage. Symptoms resolved, and she was diagnosed and treated for migraine and discharged home.

**Figure 2.**
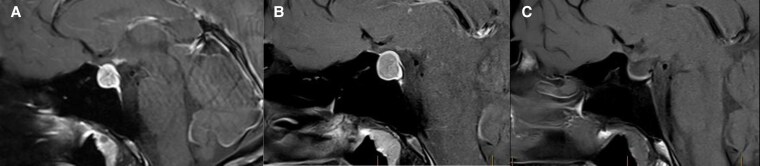
Figure depicts a series of postcontrast T1 MRI images in the sagittal plane showing the evolution of the pituitary lesion. The left-most picture shows the macroadenoma at time of diagnosis in January 2023. The middle image shows the interval increase in size in August 2023. The image on the right side demonstrates complete resolution of the image by February 2024 with possible interval development of a partial empty sella.

Cabergoline was ceased after a 12-month duration and the patient was consented for transsphenoidal surgery; however, this did not occur, as a preoperative MRI revealed complete resolution of the pituitary lesion (Fig. 2). Two months later in February 2024, repeat testing for CD was arranged. Four serial midnight salivary cortisol samples and a 24-hour urinary free cortisol demonstrated resolution of hypercortisolism (see Table 1). Imaging was reviewed, with multidisciplinary meeting consensus that there was no radiological evidence of residual tumor. The etiology was discussed with the possibility of occult apoplexy entertained. However, without evidence of T1 hyperintensity on MRI to suggest recent hemorrhage, consensus was that this was unlikely.

## Diagnostic Assessment

Results from investigations are shown in Table 1. Sequential MRI imaging is depicted in Fig. 2, panels A-C.

## Treatment

Cabergoline was commenced for treatment of symptomatic hyperprolactinemia. Emergence of biochemical and clinical hypercortisolemia occurred months after initiation of therapy.

## Outcome and Follow-Up

A surveillance MRI was performed in May 2024 and repeat midnight salivary cortisol measurement was performed in April, July, and December 2024, which demonstrated continued remission of disease. Oligomenorrhea and intermittent acne persisted following remission, but there was no further weight gain or galactorrhea. In a recent systematic review of spontaneously resolving CD, 39% of patients had recurrence of disease [3]. The patient will therefore require close surveillance to monitor for clinical signs of hypercortisolism.

## Discussion

This case of spontaneous resolution of CD is one of only 23 cases described in the literature [3]. This case has a number of interesting aspects. First, the clinical manifestation that led to medical attention was a trivially elevated prolactin which resulted in galactorrhea. Second, the full, if abbreviated, course of CD could be observed from start to end, and a wide variety of dynamic tests were employed in the pursuit of its demonstration. A third interesting feature was the mysterious resolution of the disease, with no trace of biochemical or radiological disease.

Harvey Cushing documented the first spontaneously resolving case of his disease with patient “Minnie G,” treated with subtemporal decompression to treat intracranial pressure in the 1910s [4]. She showed classic symptoms but was discharged in a “not improved condition,” surviving until 1958, suggesting a presumptive cure. Recent discussions suggest her resolution may reflect pituitary apoplexy or align more with Cushing syndrome than the disease itself [5].

CD is the most common form of endogenous hypercortisolism [6]. It is distinguished from Cushing syndrome by virtue of its origin at the pituitary. Macroadenomas, as seen in this case, account for less than 10% of cases of CD [1]. Diagnosis of CD is achieved in sequence by first objectively demonstrating hypercortisolism, and then by localization to the pituitary gland as the source [6]. In this case, the diagnosis was made in accordance with best practice [7], with clinical assessment and the employment of 3 screening tests (DST, 24-hour urinary free cortisol, and midnight salivary cortisol × 3). Late-night salivary cortisol has been reported to have a sensitivity and specificity of 97% and 97.5% respectively (although this is dependent on assay used) [7]. ACTH-dependence was demonstrated by way of dynamic endocrine testing. Although inferior petrosal sinus sampling (IPSS) is the gold standard for diagnosis of CD, international consensus is that patients with ACTH-dependent hypercortisolism and with lesions ≥10 mm on MRI do not require IPSS [7].

Definitive treatment of CD is transsphenoidal surgery at an experienced pituitary center with success rates approaching 80%, although up to 30% of patients will require additional treatments [6]. Where surgery is contraindicated or otherwise not feasible, medical treatment consists primarily of 3 classes of treatments: inhibitors of adrenal steroidogenesis (ketoconazole, osilodrostat, metyrapone, mitotane and etomidate [off-label]); therapy directed at the pituitary gland such as somatostatin receptor analogues (pasireotide) and dopamine agonists (cabergoline); and glucocorticoid receptor antagonists (mifepristone). There is no consensus on a preferred agent for medical management of CD [8]. A recent meta-analysis showed that medical treatment for CD induces disease control in less than 50% of cases, and showed similar efficacy between cabergoline, pasireotide, and ketoconazole [9]. There are no society guidelines on the optimal surveillance strategy for patients with spontaneously resolving CD.

In this case, cabergoline treatment was initiated for treatment of symptomatic hyperprolactinemia, and not as primary treatment for CD. However, cabergoline (at doses of 0.5-7 mg weekly orally) is an off-label treatment for CD that decreases tumor volume in up to 50% of cases of patients evaluated in small, prospective, observational trials [9, 10]. There are multiple mechanisms by which cabergoline may cause tumor shrinkage in prolactinomas (and by extension, corticotropin-secreting tumors), predominantly via blocking adenylyl cyclase, which in turn inhibits cyclic AMP, leading to cellular apoptosis [11]. A large prospective study and another, smaller, retrospective study both concluded that about one-quarter of CD patients have a significant clinical response to cabergoline treatment [12, 13]. However, in one prospective study, no significant clinical value was observed with its use [14]. Most trials using cabergoline monotherapy are single-center, nonrandomized, open-label trials but meta-analysis of the available data suggests that patients with milder hypercortisolism may benefit most [10]. In this case, symptoms and signs of CD evolved during cabergoline therapy, diminishing the likelihood of its role in the resolution of disease in this case.

Subclinical and overt pituitary tumor apoplexy has been postulated to be the most common cause of spontaneous remission of CD [3]. Cabergoline therapy [15, 16] and certain endocrine dynamic stimulation tests [17] have been implicated in pituitary apoplexy in macroadenomas. In this study a 4-mg IV DST and a CRH stimulation test were conducted within 3 weeks of the presentation with headache, nausea, and vomiting. A previous study has demonstrated that high T1 signal intensity (a radiological clue of recent hemorrhage) is visible on MRI 5 months following pituitary apoplexy [18]; however, this was not present in this case. Moreover, there was no radiological evidence of hemorrhage on CT imaging of the brain at the time of presentation. However, it should be noted that the sensitivity of CT in detecting pituitary apoplexy is not well established, with previous studies demonstrating a sensitivity of 21% to 46% [19]. Consensus following discussion at a multidisciplinary conference at an experienced pituitary center was that apoplexy was unlikely to have responsible for resolution of disease.

Another proposed mechanism for spontaneous resolution of CD is a change in phenotype from functioning to nonsecretory adenoma [3]. Transformation of the secretory profile of pituitary adenomas is rare, with evolution of prolactinoma to GH-secreting tumor being the most common phenotype, owing to a shared lineage of somatotrophs and lactotrophs (somato-mammotroph progenitor cells) [20]. Other types of plurihormonal pituitary adenomas are extremely rare [20, 21].

The emergence of minor clinical androgen excess with intermittent acne coincided with cessation of the COCP (prescribed for contraception), raising the possibility of undiagnosed polycystic ovarian syndrome. However, the absence of biochemical hyperandrogenism and other features makes this less likely and could not explain the convincing biochemical evidence of CD.

Strengths of this case include longitudinal radiological and biochemical data demonstrating emergence and resolution of CD. Limitations of this case report include absence of histopathological confirmation of disease and the lack of a definitive explanation for induction of disease remission.

## Learning Points

- The signs and symptoms of CD can be subtle.
- Extensive investigation is required to demonstrate ACTH-dependent hypercortisolism.
- Dopamine agonism has been used as a treatment for CD with variable rates of success.
- Pituitary apoplexy is a cause of spontaneous disease remission in CD.

## Contributors

All authors made individual contributions to authorship. S.R. and K.B. were involved in the diagnosis and management of the patient and manuscript submission. A.O. was responsible for manuscript drafting and amendments, collation of data and imaging. All authors reviewed and approved the final draft.

## Data Availability

Data sharing is not applicable to this article as no datasets were generated or analyzed during the current study
